# Living arrangements, household registration status and depression among older adults in mainland China

**DOI:** 10.1186/s12889-026-26765-y

**Published:** 2026-02-27

**Authors:** Yiqi Wangliu, Ji-Kang Chen

**Affiliations:** 1 School of Ethnology and Sociology, Yunnan University, Kunming, China; 2 Department of Social Work, T.C. Cheng Building, United College, The Chinese University of Hong Kong, Shatin, New Territories, Hong Kong, Hong Kong SAR China

**Keywords:** Living arrangements, Depression, Household registration status, Older adults

## Abstract

**Background:**

Depression constitutes a substantial psychological issue among older adults in China. Traditional Chinese culture emphasizes family care for older adults; however, modernization has increased institutional care. This study aimed to examine the association between living arrangements and depression among older adults in China while considering the moderating effect of household registration status (hukou) due to rural–urban disparities in social resources.

**Methods:**

We utilized Chinese Longitudinal Healthy Longevity Survey 2018 data involving 10,089 older adults. Depression was assessed utilizing the 10-item Center for Epidemiologic Studies Depression Scale. Living arrangements included living alone or with a spouse, with children, and in institutions. The household registration status was urban or rural. A hierarchical multiple regression analysis was performed to investigate the direct and moderating effects.

**Results:**

Older adults living with children were less depressed than those living alone or with a spouse. Older adults staying in institutions had fewer depressive symptoms. Rural older adults experienced higher levels of depression than their urban counterparts. The household registration status played a role in the relationship between living in institutions versus living alone or with a spouse and depression, where urban areas showed a significant positive effect.

**Conclusion:**

The results are consistent with the convoy model of social relations, social integration theory, and the stress-buffering hypothesis, which explain how social resources and living arrangements can lessen depression among the older adults. Living in urban areas provides more opportunities for social networks and resources, contributing to better mental health. The present work serves to inform urban and rural policies with regard to increasing social support and decreasing ‍‌depression.

## Introduction

Depression is one of the most widespread chronic illnesses affecting older people in China [1]. A recent meta-analysis has shown that the rate of depression has gone up to 7.9% [2]. Hence, it is crucial to concentrate on depression of the older adults in China.

In China, the rapid modernization has greatly changed the pattern of intergenerational living and family relationships. At the same time, it has had deep and multidimensional impacts on the mental health of older adults [3]. The most significant change here is that the working-age population has migrated from rural to urban areas on a large scale, and the older adults left behind in the rural hometowns are therefore called "empty-nest" older adults. This massive demographic shift has significantly altered the traditional family structure where members lived together and hence disrupted the long-time caregiving practice based on proximity and daily interaction. Furthermore, urbanization has led to smaller nuclear families, more women in the labor market, and faster population aging, thereby placing greater pressure on the family care model. The varied living arrangements of the older adults, such as the traditional multi-generational household, living alone, or in a nursing home, each have different psychological effects because they influence the level of social support, the strength of intergenerational relationships, and the amount of freedom the older adults get to ‍‌experience.

In ‌ old times, Chinese seniors still largely depended on family care, a system that was deeply ingrained in the cultural value of filial piety (xiao), which is a way of showing obedience, respect, love, and giving physical care to the elders in the family [4]. At the basis of older adults’s care in Chinese history is this family caregiving paradigm, which has been continuously supported by Confucian ethics that make family responsibility a moral duty. Nevertheless, the clash between modernization and traditional filial piety has created a complicated situation. It is true that the central values of xiao, such as reverence for the older adults, are still very much a part of people’s consciousness, but their real-life applications have changed. One example is the extensive rural-to-urban migration that has compelled adult children to develop new ways of demonstrating filial piety, which, in most cases, is done by sending money instead of being physically present. This change has created a discrepancy between the desire to provide home care and the situation of being far apart, thus causing emotional pain for both the older adults (who may feel neglected) and their children (who may feel guilty). In addition, the Chinese government’s support for care-institution programs has emerged as a measure for "Three-No" elders (those who are without family members, income, or the ability to work) [5]. However, this option is often looked down upon. Even seniors who choose residential care due to being far away from their children may face the lingering misconception that placing parents in a nursing home or a retirement facility (defined as places where professional care is given [3, 6]) is "unfilial." This tension highlights the ongoing struggle to reconcile modern practice with traditional values.

Earlier studies have explored different aspects of the living arrangements of the older adults being alone, with family, or in an institution and their health impact, which brought about different conclusions. On one hand, some researches convinced that living together may lead to disputes between the old and the young, as a result, the health of older adults might be at risk [7]. Contrarily, other researches argued that living together may be beneficial to the health of older adults as it provides them with the emotional and material support from the family members [8]. It has been found that, among elders, death and depression rates in institutions have been increased [9, 10]. On the other hand, living alone has also been linked with several negative health issues, such as depression, cardiovascular disease, and dementia ‍‌[11, 12].

Recently, ‍‌ researchers utilizing the 2018 CLHLS dataset have focused on how living arrangements impact the depression of older adults Chinese people. Xie et al. argued that there is a direct relationship between living arrangements and depressive symptoms [13]. Then, Jia et al. went further with their study and pointed out that activity participation is a mediator [14]. These studies have significantly deepened the knowledge of the psychosocial factors that explain the link. However, they do not consider the influence of family registration (hukou) which is a major institutional factor determining resource availability in ‍‌China. China's household registration system (hukou) has resulted in significant social and economic divides between rural and urban residents since the 1950 s [15]. Older adults people in rural areas normally see a lower level of access to state-subsidized goods and health care than the urban older adults, and also receive less social support and have fewer opportunities for social interaction [15, 16]. This paper addresses the issue of depression among older adults in rural area who are often neglected. It examines how the effect of living alone on depression changed based on a person's hukou status, shifting the focus from individual-level mediators to broader sociostructural factors. Through the institutional dimension, we want to reveal the complex picture of the mental health situation among the older adults in present-day ‍‌China.

### Living arrangements and depression among older adults

The Convoy Model of Social Relations [17] offers a theoretical framework for examining social relationships among individuals [18]. This model proposes that close social relationships form a "convoy" of diverse resources that provide support to individuals throughout their lives. Family sociologists have suggested that co-residence constitutes one of several social transfers originating from the kin group and directed toward older parents. Families with multi-generations may function as a "convoy" [17] that aids older adults in effectively overcoming life challenges by providing financial and physical support. Older adults in such family settings have relatively easy access to financial capital (household income, loans, investments) and embodied cultural capital (knowledge and good practices acquired through exposure to extra-familial networks). Therefore, their health may be improving. However, individuals living alone may possess limited social resources. With limited resources, they must strive to fulfill their social and emotional needs through relationships outside their household, which can be challenging and may result in adverse health outcomes.

Previous studies demonstrated inconsistent results regarding the relationship between older adults living in community settings and those in institutional care. In developed countries, institutionalization is frequently regarded as the last resort for families who have exhausted their healthcare resources in providing for older adults experiencing progressive functional or cognitive deterioration [6]. Consistent with this, studies from developed countries, including America and Japan, demonstrated that older adults in institutional care exhibited lower levels of happiness than their community-dwelling counterparts [19]. However, the comparative psychological status of institutionalized and community-dwelling older adults in China is under-documented. A few studies have examined the psychological well-being of institutionalized and non-institutionalized older adults in China, primarily focusing on quality of life. These studies consistently demonstrate that older adults in institutional care exhibit higher levels of quality of life than those living in the community [8, 20]. It is plausible that the older adult residents in institutions may benefit substantially from increased social interactions, activities, and perceived support available within institutional settings.

The association between depression and living arrangements among older groups living in the community has been previously examined across various societies, with results differing across cultures. Studies in Western countries have primarily focused on the impact of living alone and the effect of living with others on depressive symptoms. A study conducted in America demonstrated that older adults living alone exhibited higher levels of depression than those living with other people [20]. Similarly, a Finnish study demonstrated that individuals living alone or with others are more susceptible to depressive disorders than those living with a spouse [21]. However, the structure of living arrangements in Asia is more diversified. Most previous studies have indicated that older adults living with children overseas exhibited higher depression levels [22]. For instance, in Singapore, those living alone and those living with their children exhibited higher depression levels [23]. In Korea, older adults living alone, with an unmarried child, or with grandchildren are more susceptible to depressive symptoms [7]. In Thailand, having a child living in the same district was associated with a higher likelihood of depression among older adults [24]. However, another study demonstrated an inverse relationship, indicating that living with children is a protective factor against depression [25]. In China, living arrangements were significantly associated with depression among older adults. For instance, older adults living alone or in institutional care have higher depression levels than older adults living with their families [26]. Silverstein et al. [27] drew similar conclusions; they reported that older adults living with their children might exhibit fewer depressive symptoms. Given traditional cultural values in China and the prevalence of single-child families, parental depression may be more pronounced in China [27]. Following the implementation of China's universal two-child policy in 2015 [28], parents have encountered increased caregiving burdens.

### The moderating role of household registration status

Social Integration Theory highlights the significance of an individual's connection with resources [29]. When individuals receive rich social resources, the negative influence of stressful events is mitigated. This is consistent with the stress-buffering hypothesis, which argues that social resources can reduce the negative effects of stressors on health [30]. Particularly in urban areas, individuals may have access to abundant resources, and with abundant resources, older adults may find it easier to navigate stressful events. However, in rural areas, where access to resources may be limited, the capacity to manage stressors could be diminished accordingly.

Furthermore, extensive studies have demonstrated rural–urban differences in health and healthcare resources among older adults [16, 31]. Regarding psychological health, depression is considerably more common among older adults in rural areas and might result in higher suicide rates [15]. Therefore, this study aimed to examine the disparities between rural and urban living arrangements and their correlation with depression among older adults.

As previously stated, the household registration status often limits rural older adults’ access to healthcare services and resources and reduces social support and participation opportunities [15, 16]. Most older adults in rural areas depend on their adult children for financial and instrumental support when necessary [16]. Empirical studies demonstrate that living alone is associated with higher levels of depression in rural areas [32]. Based on these findings, we hypothesized that living without an adult child may be more detrimental to the psychological health of rural older adults, specifically based on depression, than for those older adults living in urban areas.

### This study

This study aimed to examine the relationship between living arrangements and depression among older adults and investigate whether their household registration status serves as a moderator in this relationship. Based on the Convoy Model of Social Relations [17], older adults living with family members have more access to social resources. Therefore, we hypothesized that older adults, those living alone or with a spouse and in institutions, might experience higher levels of depression than those living with adult children (H1).

Regarding the moderating effect of household registration status, on the basis of social integration theory [29] and the stress-buffering hypothesis [7], we hypothesized that if older adults live in urban areas, the negative influence of living alone or with a spouse and institutions might be mitigated (H2).

## Methods

### Research design

This study employed the dataset of the Chinese Longitudinal Healthy Longevity Survey (CLHLS). This is a long-term household follow-up survey of adults aged ≥ 65 years and their adult children in 23 provinces in China. This dataset collected data on socioeconomic backgrounds, health characteristics, and social participation of older adults. The baseline survey was conducted in 1998, followed by seven follow-ups. This dataset has been demonstrated to be reliable and valid, and thousands of papers have been published based on this dataset. This study applied the most recent follow-up survey in 2018 after excluding missing data. This study included 10,089 older adults.

### Measures

Based on previous research that used the CLHLS dataset [18, 33], we selected a set of variables for the data analysis (Table 1).

**Table 1:** Descriptive statistics of the sample (*n* = 10,089)

Variables	Description and Coding	N (%)/Mean (*SD*)
Independent variables		
Living arrangements (reference)	Alone or spouse	53.72%
	With adult children	42.54%
	In institutions	3.74%
Dependent variables		
Depression	Range = 0–30	22.56 (6.24)
Control variables		
Age	Range = 58–124	90.71 (11.42)
Gender	Male = 1	43.62%
	Female = 0	56.38%
Marital status	Partnered = 1	41.08%
	Non-partnered = 0	58.92%
Household registration status	Urban = 1	27.5%
	Rural = 0	72.5%
Years of schooling	Range = 0–16	3.54 (6.68)
Self-rated health	Range = 0–9	3.02 (1.72)
ADL	Range = 0–18	16.72 (2.78)
Ethnicity	Han = 1	94.05%
	Others = 0	5.95%
Social engagement	Range = 0–16	11.04 (7.61)

*SD* Standard deviation

### Dependent variable (Depression)

Depression is the dependent variable in this study. It is assessed with the 10-item Center for Epidemiologic Studies Depression Scale (CES-D-10). This scale is frequently used to assess older adults’ depression in China, with good reliability and validity. There are ten items in CES-D-10, and the score of each item is divided into four grades: 0 = "rarely" to 3 = "most of the time." Two positive items (happy and hopeful) in the CESD-10 have been reverse-coded. The range of the CES-D-10 score is from 0 to 30. Higher scores indicate severe depression. The Cronbach’s alpha of CES-D-10 was 0.810, which indicated a reasonable internal consistency level.

### Independent variable

The living arrangement is an independent variable in this study. We divided the living arrangements into three categories: living alone or with a spouse, living with children, and living in an institution; living alone or with a spouse was included as a reference item.

### Moderator

Household registration status was coded as “1 = urban” and “0 = rural.”

### Controls

Based on previous studies [27, 34], sociodemographic factors included in this study were age, education, marriage, ethnicity, self-rated health, years of schooling, and activities of daily living (ADL) and social participation. Table 1 presents the general information on these variables. For instance, ethnicity was recorded as “1 = Han” and “0 = other minorities.” Marital status was divided into “1 = have a partner” and “0 = do not have a partner.” Self-rated health was assessed by “How do you rate your health at present?” and years of schooling were assessed by asking, “How many years did you attend school?” Six ADL items included dressing, grooming, toileting, bathing, ambulation, and transfer. The participants indicated whether they provide support with these tasks for older adults. The responses ranged from none (0) to a lot (3). Based on the social exchange theory, social participation includes activities organized by the community or neighborhood [21]. In a recent study [35], the following question was asked: “Do you perform the following activities regularly?” Activities included outdoor activities, playing cards and Mah-jong, and organized social activities. Social activity was assessed by the participation frequency, which was classified as “1, almost every day; 2, not daily, but at least once/week; 3, not weekly, but at least once/month; 4, not monthly, but sometimes; 5, never” by the question. We reverse-coded each item from 0 (never) to 4 (almost every day) and added the score of each activity to present the total social participation scores. The higher the score, the higher the level of social participation.

### Data analysis

The statistical software Stata was utilized for data analysis. Descriptive statistics were computed to summarize the sample characteristics and the variables under examination. We‍‌ did a hierarchical multiple regression analysis to examine the direct and moderating effects of household registration status on depression. At first, the control variables were taken along as covariates in the model. Then, in the second step, the centered independent variables (living arrangement) and the centered potential moderator (household registration status) were added to test Hypothesis 1. In the last step, the interaction terms between the living arrangement and the potential moderator were introduced to test Hypothesis 2. Using the approach suggested by Smith et al. [36], the analysis determined if the model with the interaction term significantly explained more variance than the model without it. A significant increase in explained variance, together with a statistically significant regression coefficient for the interaction term, was considered evidence of a moderating ‍‌effect.

### Descriptive statistics

Table 1 presents the results of descriptive analysis. The mean score for depression was 22.56 (standard deviation [SD] = 6.24), with a possible range from 0 to 30. The percentages of living alone or with a spouse, with children, and living in institutions were 53.72%, 42.54%, and 3.74%, respectively. The percentage of older adults living in rural areas was approximately 72.5% compared to those in urban areas, at 27.5%.

The mean age of the participants was 90.71 years old (SD = 11.42). Additionally, 56.38% of the participants were women, and 41.06% were currently partnered. Most of them were from Han (94.05%). The average years of schooling was 3.54 (SD = 6.68). The average of self-rated health and ADL were 3.02 (SD = 1.72) and 16.715 (SD = 2.78), respectively.

### Direct and moderating effects of perceived community support

Based on Table 2, the Model 1 analyses revealed significant associations between depression and sex (*β* = -.527, *p* <.001), marital status (*β* = -.819, *p* <.001), years of schooling (*β* = –.056, *p* <.001), ethnicity (*β* = −.720, *p* <.05), ADL (*β* = −.118, *p* <.001) self-rated health (*β* = −2.773, *p* <.001) and social engagement (*β* = −.102, *p* <.001).

In Model 2, the main effects revealed that compared with older adults living alone or with a spouse, individuals living with adult children exhibited lower levels of depression (*β* = −.811, *p* <.001). However, individuals living in institutions exhibited lower levels of depression than those living alone or with a spouse (*β* =.−671, *p* <.05). Additionally, compared with those older adults living in rural areas, those living in urban areas were more likely to experience lower depression (*β* = −.619, *p* <.001).

In Model 3, the interaction effects indicated that household registration status moderated the relationship between the living arrangements (living in institutions versus living alone or with a spouse) and depression. The type of household registration moderates the effect of living arrangements (living in institutions versus living alone or with a spouse) on depression. However, household registration status did not moderate the relationship between living arrangements (living with children versus living alone or with a spouse) and depression (Table 2).

**Table 2:** Effects of household registration status on the associations between living arrangement and depression: hierarchical regression estimates (*n* = 10,089)

	Depression		
Predictor variables	*Model 1*	*Model 2*	*Model 3*
Controlled			
Age	−0.009	−0.001	0.0018
Gender	−0.527***	−0.596***	−0.597***
Marital status	−0.819***	−1.164***	−1.163***
Years of schooling	−0.056***	−0.036***	−0.035*
Ethnicity	−0.720*	−0.768***	−0.754*
ADL	−0.118***	−0.138***	−0.136***
Self-rated health	−2.773***	−2.766***	−2.766***
Social engagement	−0.103***	−0.095***	−0.095***
Independent Variable			
Living with adult children		−0.811***	−0.817***
Living in institutions		−0.671*	−0.474*
Moderator Variable			
Household registration status		−0.619***	−0.695***
Interaction			
Living with adult children x Household registration status			0.013
Living in institutions x Household registration status			1.741*
F	220.84***	330.19***	368.04***
R^2^	0.19	0.26 (0.07)	0.28 (0.02)

R^2^ = R square; *** *p* <.001, ** *p* <.01, * *p* <.05

**Fig. 1:**
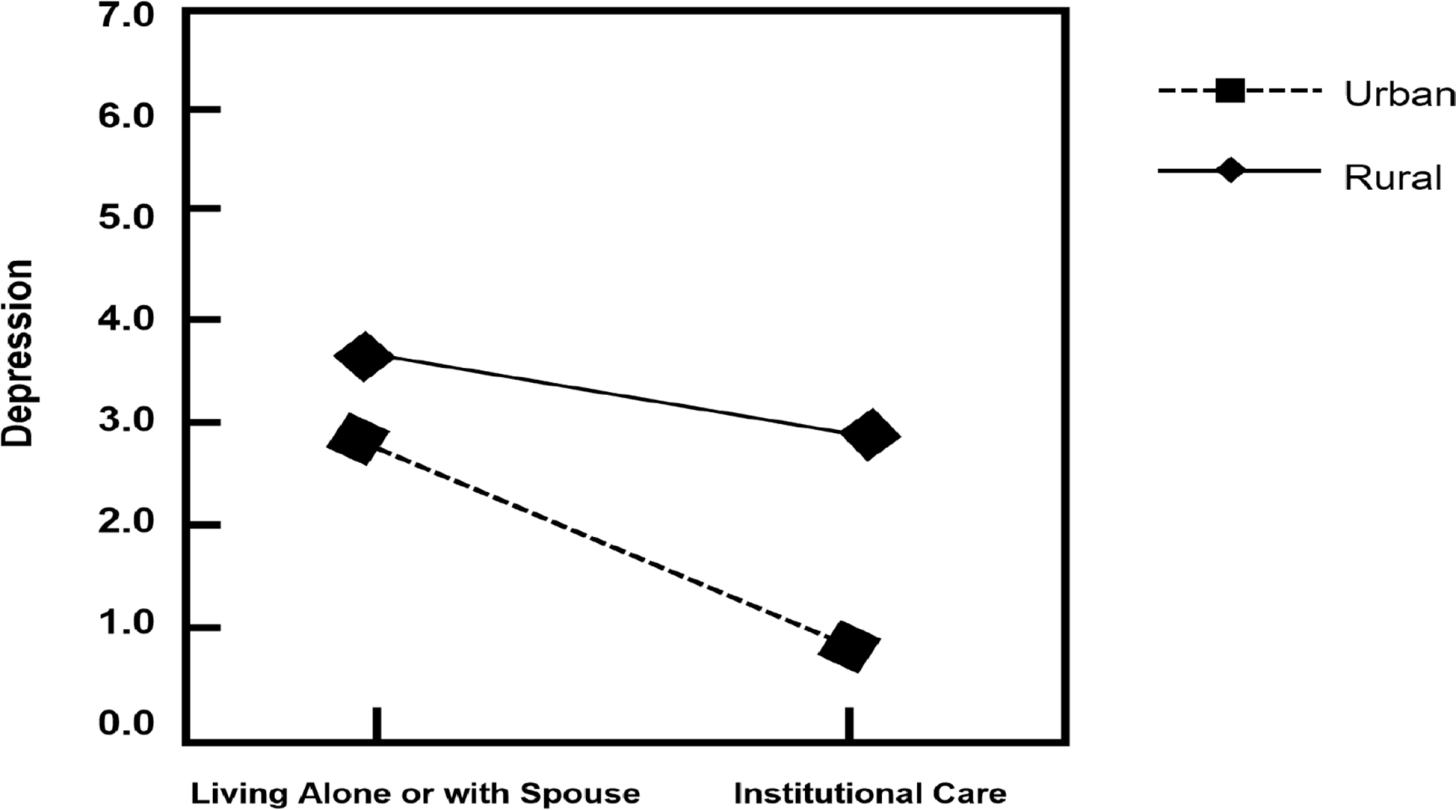
Simple slope tests on the relationships between household registration status, living arrangements and depression

To further interpret the significant interaction, we conducted simple slope tests [2]. In Fig. 1, the x-axis shows living arrangements of living alone or with spouse and in residential care, and the Y-axis represents depression. In Fig. 1, for urban group, the difference of depression levels between living alone or with spouse and institution is significant (*p* = 0.001) but the difference in rural group is not (*p* = 0.07). These simple slope test results revealed significant differences in the slopes of urban or rural groups in the relationship between living arrangements and depression among older adults.

## Discussion

This study demonstrated that older adults living with adult children exhibited significantly lower levels of depression than those living alone or with a spouse. However, individuals living in institutions exhibited lower levels of depression than those living alone or with a spouse. Furthermore, older adults living in urban areas experienced lower depression levels than those in rural areas. The interaction effects indicated that household registration status moderated the relationship between living arrangements (living in institutions versus living alone or with a spouse) and depression. Specifically, institutional residence is associated with significantly lower depression only among urban older adults.

Much‍‌ of the main results are consistent with some of the previous research that older adults living with their adult children report less depression than those living by themselves, and people living in rural areas have more depressive symptoms, which are usually linked to different social and health care resources in these areas [15, 16]. Following these trends on a large, nationally representative dataset from 2018 shows that these issues persist despite social changes in China. Nevertheless, the main outstanding point of this study is to reveal the moderating role of household registration status, which greatly impacts the mental health consequences of being institutionalized. To be more specific, the positive impact of living in an institution on depression is strong for older urban residents, which means that the benefits of formal care are not the same for people living in different geographical or administrative divisions [8, 20]. Thus, hukou indicates more than just resource inequality; it serves as a significant contextual factor that moderates the association between living arrangement and mental health. This understanding sheds light on how structural factors influence the capacity and effectiveness of alternative care models in old ‍‌age.

Moreover, ‍‌ this research has shown that older adults people living in cities have less depression than those living in the countryside. Cities are usually equipped with better facilities such as healthcare, social services, and recreational activities that might be the reasons why older adults individuals from an urban area have better psychological outcomes [15, 16]. In urban settings, a lot of older people get to be part of different social networks and social activities, which are very necessary for their psychological ‍‌health. Moreover, the fact that living in an institution is related to a significantly lower level of depression only in the case of older adults living in urban areas. The findings aligns with a previous research highlighting disparities in the quality of institutional care between urban and rural settings. For example, urban facilities often have a more organized mental health support system [15], whereas rural areas may struggle due to limited resources, hindering their ability to improve mental health outcomes through institutional care ‍‌[20].

This study observed that living with adult children and residing in institutions were associated with lower levels of depression among older adults, which is consistent with the predictions of the Convoy Model of Social Relations. According to this model, close social ties serve as a protective "convoy" throughout life. While we did not directly measure perceived social support or relationship quality, the observed mental health advantages among those co-residing with children or in institutional care may reflect greater access to social interactions and structured activities, which are potential components of convoy-like support systems. Our research found that the older adults who live with their adult children or are put in care homes, tend to have less depression. This goes along with the idea of the Social Relations Convoy Model. The model claims that close social bonds are like a protective "convoy" that goes with you through life. Although we didn't test for perceived social support or relationship quality, the better psychological state of those living with their children or receiving institutional care might indicate that they have more social interaction and planned activities – these could be elements of support systems similar to a "convoy".

In‍‌ addition, the study results also agree with the prediction of social integration theory and the stress-buffering hypothesis, which both assume that a good social environment can reduce a person's mental trouble [15]. The findings indicate that urban areas, with their more tightly woven service networks and greater variety of social opportunities, might increase the positive effects of some living arrangements, especially the institutional residence one. Yet, as we did not directly measure variables such as loneliness, emotional support, or perceived social cohesion, these explanations are only our assumptions. It would be advisable for subsequent studies to use recognized and reliable scales of interpersonal relationships and subjective belonging in order to tease out the mechanisms behind these theories more ‍‌precisely.

This‍‌ study is not without its limitations. Firstly, the study's reliance on the evaluation of self-reported depressive symptoms could introduce bias. Moreover, using social engagement and living arrangements measures might not be able to fully encapsulate the complexity of these concepts. Additional studies should think about incorporating more detailed and objective methods for measuring the variables involved. Secondly, the present study's cross-sectional nature did not allow for the establishment of causal relationships. Therefore, longitudinal studies might be necessary to unravel the causal relationships between living arrangements, household registration status, and depression. Thirdly, although there is an interaction of household registration status in this study, the dynamics that lead to these interactions were not explored. It would be valuable to study the exact mechanisms through which household registration status influences the relationship between living arrangements and ‍‌depression. Data‍‌ were analyzed without the use of sample weights that are usually recommended when working with the CLHLS dataset to consider complex survey design and obtain nationally representative estimates and when sample weights are not used, the findings may thus not be broadly generalizable to the population of older adults in China [37]. Lastly, we have recognized the possibility of unobserved heterogeneity and have incorporated sensitivity analyses in the revised manuscript. To be specific, we retraced the model using “living alone” and “living with spouse” as separate reference groups. The main results (i.e., the beneficial impact of intergenerational co-residence) were still in line, without any qualitative changes in effect sizes or significance. We also factored in marital status and social support in the main model, which somewhat explains differences in emotional interaction between the two ‍‌groups.

This‍‌ study also carries with it some very important practical implications. For one, policies that emphasize family care and local community support programs should be considered as they might be able to alleviate depression among the older adults. Furthermore, since the household registration status has a moderating effect, policies should be designed to meet the particular requirements of urban and rural older adults conforming to the local conditions. Two, healthcare professionals may come up with specific treatments for depression in the older adults. As an example, finding ways to enhance social involvement could be a particularly good solution for older adults people who are living by themselves or in care homes. Besides this, healthcare professionals can collaborate with families and communities to augment the social networks of very old people thus lowering their risk of depression. Community organizations have a responsibility to foster social interaction and provide recreational opportunities for the older adults. For example, they can motivate older adults people to take part in group activities, participate in exercise classes, and attend educational workshops. Such programs can significantly contribute to the older adults social integration and help them overcome the feeling of ‍‌loneliness. Moreover, there might be a risk of selection bias regarding the older adults living in institutions, as those who decide or are allowed to enter institutions may be quite different in some aspects from the ones who stay in the community (for instance, they may have different health conditions beforehand, family support, or be different in terms of socioeconomic resources). Although increasing institutional care might appear to be a solution for urban older adults, efforts should be made to tackle the structural problems that limit the possibility of the most vulnerable groups to access this kind of care, such as the issue of affordability and accessibility, rather than simply assuming that institutionalization per se will reduce ‍‌depression.

## Conclusion

This research pointed out key elements that contribute to depression among older adults people, with a major emphasis on how living conditions and household registration affect the issue. The results were in line with the theoretical frameworks and served as a good base for implementing practical measures to improve older adults people's health. Still, it indicates that additional studies should be conducted to explore the intricate social factor-mental health relationships in later life and to better understand the ‍‌situation.

## Data Availability

The data in this analysis are available from authors upon reasonable request.
